# Hemoglobin-Improved Protection in Cultured Cerebral Cortical Astroglial Cells: Inhibition of Oxidative Stress and Caspase Activation

**DOI:** 10.3389/fendo.2017.00067

**Published:** 2017-04-10

**Authors:** Fatma Amri, Ikram Ghouili, Marie-Christine Tonon, Mohamed Amri, Olfa Masmoudi-Kouki

**Affiliations:** ^1^University of Tunis El Manar, Faculty of Sciences of Tunis, UR/11ES09 Laboratory of Functional Neurophysiology and Pathology, Tunis, Tunisia; ^2^INSERM U1239, Laboratory of Neuronal and Neuroendocrine Communication and Differentiation, Institute for Research and Innovation in Biomedicine (IRIB), University of Rouen Normandie, Mont-Saint-Aignan, France

**Keywords:** hemoglobin, oxidative stress, astrocytes, apoptosis, cell protection

## Abstract

Oxidative stress plays a major role in triggering astroglial cell death in diverse neuropathological conditions such as ischemia and neurodegenerative diseases. Numerous studies indicate that hemoglobin (Hb) is expressed in both resting and reactive glia cells, but nothing is known regarding a possible role of Hb on astroglial cell survival. Thus, the purpose of the present study was to investigate the potential glioprotective effect of Hb on hydrogen peroxide (H_2_O_2_)-induced oxidative stress and apoptosis in cultured rat astrocytes. Our study demonstrates that administration of graded concentrations of Hb (10^−12^ to 10^−6^ M) to H_2_O_2_-treated astrocytes reduces cell death in a concentration-dependent manner. H_2_O_2_ treatment induces the accumulation of reactive oxygen species (ROS) and nitric oxide (NO), a drop of the mitochondrial membrane potential, and a stimulation of caspase-3/7 activity. Exposure of H_2_O_2_-treated cells to Hb was accompanied by marked attenuations of ROS and NO surproductions, mitochondrial membrane potential reduction, and caspase-3/7 activity increase. The protective action of Hb was blocked by the protein kinase A (PKA) inhibitor H89, the protein kinase C (PKC) inhibitor chelerythrine, and the mitogen-activated protein (MAP)-kinase kinase (MEK) inhibitor U0126. Taken together, these data demonstrate for the first time that Hb is a glioprotective factor that protects astrocytes from apoptosis induced by oxidative stress and suggest that Hb may confer neuroprotection in neurodegenerative diseases. The anti-apoptotic activity of Hb on astrocytes is mediated through the PKA, PKC, and MAPK transduction pathways and can be accounted for by inhibition of oxidative stress-induced mitochondrial dysfunctions and caspase activation.

## Introduction

Hemoglobin (Hb) is a heme protein mainly present in erythrocytes of vertebrates (1, 2). Hb is known to function as a carrier protein of oxygen (O_2_) and carbon monoxide (CO), and thus ensures transport of O_2_ to the tissues, cell oxygen-consuming, and catalysis of redox reactions (3–6). It was traditionally thought that release of Hb from erythrocytes was deleterious for the brain during intracerebral hemorrhage and trauma (2, 7, 8) and that free Hb induced a strong oxidative stress and inflammation, which were responsible for neuronal apoptosis (9–12). However, increasing evidences suggest that neurotoxicity associated to Hb derives from erythrocyte breakdown and may be accounted for iron accumulation in tissue neighboring hematomas (9, 13), rather than an accumulation of Hb molecule by itself (8, 14). In fact, iron chelators provide neuroprotection in intracerebral hemorrhage (15, 16), or in cultured astrocytes or neurons incubated with higher doses of Hb (9, 17, 18). Moreover, it has been reported that low doses of Hb maintain nitric oxide (NO) homeostasis and act as scavenger of reactive oxygen species (ROS) such as hydrogen peroxide (H_2_O_2_) (19–22) to protect cells from oxidative damages. Indeed, it has been reported that Hb prevents death of presenilin-1-deficient neurons induced by oxidative stress (23). In addition, overexpression of Hb has been found to be protective in rat models of ischemia and thereby increases neuron survival in hypoxic conditions (24). Altogether, these observations suggest that endogenous Hb could exert a strong protective effect and attenuate cellular ROS accumulation.

There is now clear evidence that Hb is expressed not only in erythroid cells but also in other cell types including neurons and glia cells (6, 25, 26). The occurrence of Hb-like immunoreactivity and Hb mRNA has been visualized in different regions of the brain, notably in the cortex, hippocampus, and cerebellum (13, 25–27). In particular, it has been shown that Hb transcript and protein levels are increased in cortical neurons and astroglial cells during the preconditioning phase of ischemia both *in vivo* and *in vitro* (28, 29). On the other hand, high concentrations of Hb mRNA and protein are found in human gliomas (30), indicating that over expression of Hb may have a role in promoting cell proliferation. An age-related decline in Hb expression in astrocytes has been found, suggesting that loss of this protein may increase susceptibility to age-related neurological disorders (31). Moreover, it has been indicated that induction of glia Hb expression contributes to reduce neuronal cell death under hypoxia conditions (24). Altogether, these results suggest that upregulation of astrocytic Hb may play a critical role in the pathogenesis of brain disorders; however, the function of endogenous astroglial Hb, which is present in much lower concentrations than found in blood, in astrocyte proliferation and/or survival is still not clear.

Since astrocytes play prominent role in the protection of neurons against oxidative injury, loss of astroglial cells may critically impair neuronal survival (32, 33). Thus, protection of astrocytes from oxidative insult appears essential to maintain brain function. Although there is clear evidence that Hb expression is upregulated in astroglial cells after traumatic brain injury and stroke (24, 29) and could exert neuroprotective activity (23, 30), a possible effect of Hb on astrocyte survival has never been investigated.

The purpose of the present study was thus to examine the potential protective action of Hb against H_2_O_2_-induced astroglial cell death and to investigate some of the mechanisms involved in this effect.

## Animals and Methods

### Animals

Wistar rats (Pasteur Institute, Tunis) were kept in a temperature-controlled room (21 ± 1°C), under an established photoperiod (lights on 0700–1900 hours) and with free access to food and water.

### Reagents

Dulbecco’s modified Eagle’s medium (DMEM), Ham F12 culture medium, d(+)-glucose, l-glutamine, N-2-hydroxyethylpiperazine-N-2-ethane sulfonic acid (HEPES), fetal bovine serum (FBS), and the antibiotic-antimycotic solution, were obtained from Life Technologies (Grand Island, NY, USA). H89, chelerythrine, fluorescein diacetate-acetoxymethyl (FDA-AM), dimethyl sulfoxide (DMSO), insulin, Triton X-100, and bovine Hb were purchased from Sigma-Aldrich (St. Louis, MO, USA). The lactate dehydrogenase (LDH; EC 1.1.1.27) assay kit was obtained from Bio-Maghreb (Tunis, Tunisia). 5-6-chloromethyl 2′-7′dichlorodihydrofluorescein diacetate (CM-H_2_DCFDA), JC-10, and 4,5-diamino fluorescein diacetate (DAF-FM) were from Molecular Probes (Eugene, OR, USA). U0126 and Apo-ONE Homogeneous Caspase-3/7 assay kit were supplied by Promega (Charbonnières, France).

### Cell Culture

Secondary cultures of rat cortical astrocytes were prepared from 1- or 2-day-old Wistar rats of both sexes as previously described (34). Briefly, cerebral hemispheres were dissected, meninges removed, and tissues collected in DMEM/Ham F12 (2:1; v/v) culture medium supplemented with 2 mM glutamine, 1‰ insulin, 5 mM HEPES, 0.4% glucose (final concentration of glucose in culture media is 4.5 g/L), and 1% of the antibiotic-antimycotic solution. The tissues were dissociated mechanically with a syringe equipped with a 25 G gage needle, and filtered through a 100-µm sieve (Falcon, Franklin Lakes, NJ, USA). Dissociated cells were resuspended in culture medium supplemented with 10% FBS, plated in 75-cm^2^ flasks (Greiner Bio-one GmbH, Frickenhausen, Germany) at the density of 17 × 10^6^ cells/mL and incubated at 37°C in 5% CO_2_/95% air. After 7–8 days, the cultures became confluent and loosely attached microglia cells and oligodendrocytes were removed by shaking the flasks on an orbital agitator (250 rpm, 18 h). Adhesive cells (mostly astrocytes) were detached by trypsinization, harvested and plated on 24- or 96-well plates at a density of 8 × 10^4^ cells/mL. All experiments were performed on 5- to 7-day-old secondary cultures and more than 98% of the cells were labeled with antibodies against glial fibrillary acidic protein (35).

### Experimental Design

**Figure 1:**
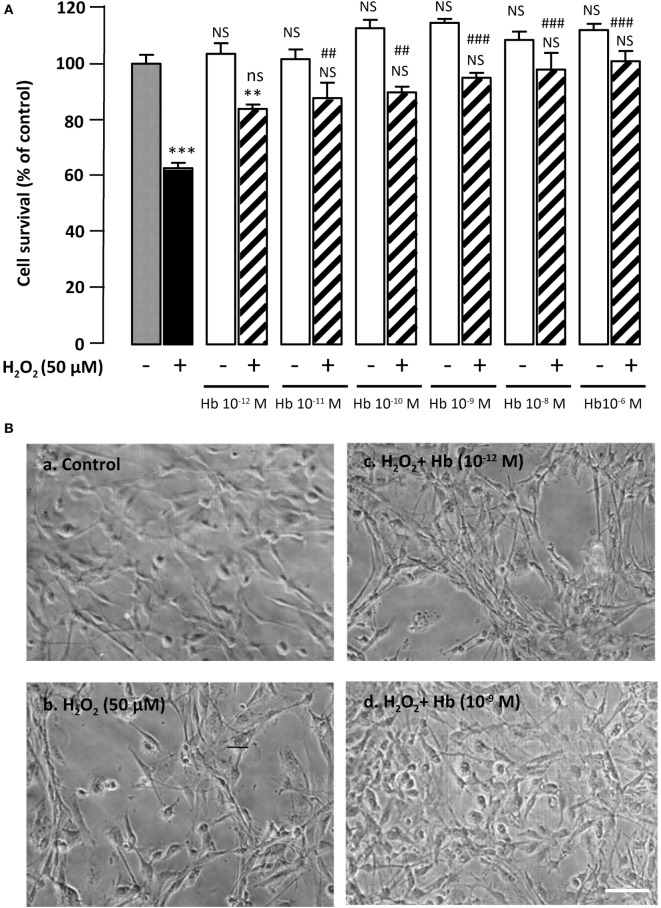
**Glioprotective effect of Hb on H_2_O_2_-induced cell death**. **(A)** Cells were co-incubated for 24 h with medium alone (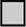) or with H_2_O_2_ (50 µM) in the absence (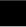) or presence of graded concentration of Hb (10^−12^ to 10^−6^ M, 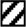). Cell survival was quantified by measuring FDA fluorescence intensity, and the results are expressed as percentages of the control. Data are means ± SEM of four independent experiments. ANOVA followed by Bonferroni’s test ***p < 0.001; NS, not statistically different from control cells (absence of H_2_O_2_ and absence of Hb). ^##^*p* < 0.01; ^###^*p* < 0.001; ns, not statistically different vs. H_2_O_2_-treated cells. **(B)** Typical phase-contrast images illustrating the effect of Hb on H_2_O_2_-induced morphological changes in cultured rat astrocytes. Cells were incubated for 24 h with medium alone (a), or 50 µM H_2_O_2_ in the absence (b), or presence of 10^−12^ M Hb (c), or 10^−9^ M Hb (d). Scale bar 100 µm.

**Figure 2:**
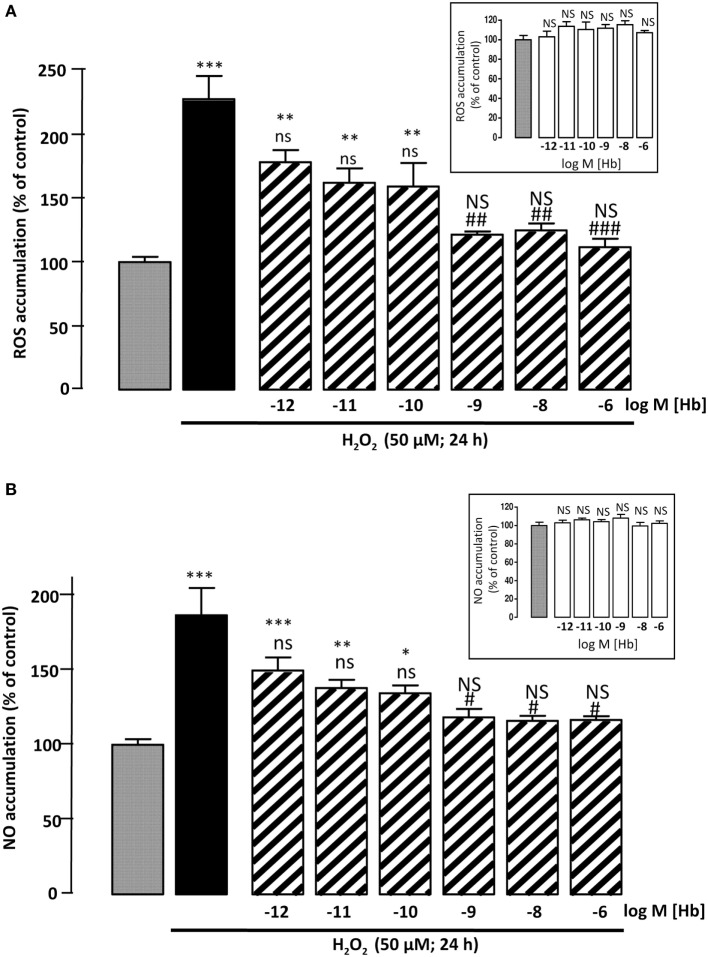
**Effect of Hb on H_2_O_2_-induced ROS and NO intracellular accumulations**. Cells were co-incubated for 24 h with medium alone (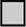) or with H_2_O_2_ (50 µM) in the absence (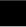) or presence of graded concentration of Hb (10^−12^ to 10^−6^ M, 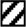). **(A)** Cellular ROS level was quantified by measurement of DCF fluorescence intensity. **(B)** Cellular NO level was quantified by measurement of DAF fluorescence intensity. Inset, effect of graded concentration of Hb (10^−12^ to 10^−6^ M; 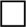) on intracellular ROS accumulation **(A)** and NO formation **(B)**. The results are expressed as percentages of the controls. Data are means ±SEM of three independent experiments. ANOVA followed by Bonferroni’s test *p < 0.05; **p < 0.01; ***p < 0.001; NS, not statistically different from control cells. ^#^*p <* 0.05; ^##^*p* < 0.01; ^###^*p* < 0.001; ns, not statistically different vs. H_2_O_2_-treated cells.

**Figure 3:**
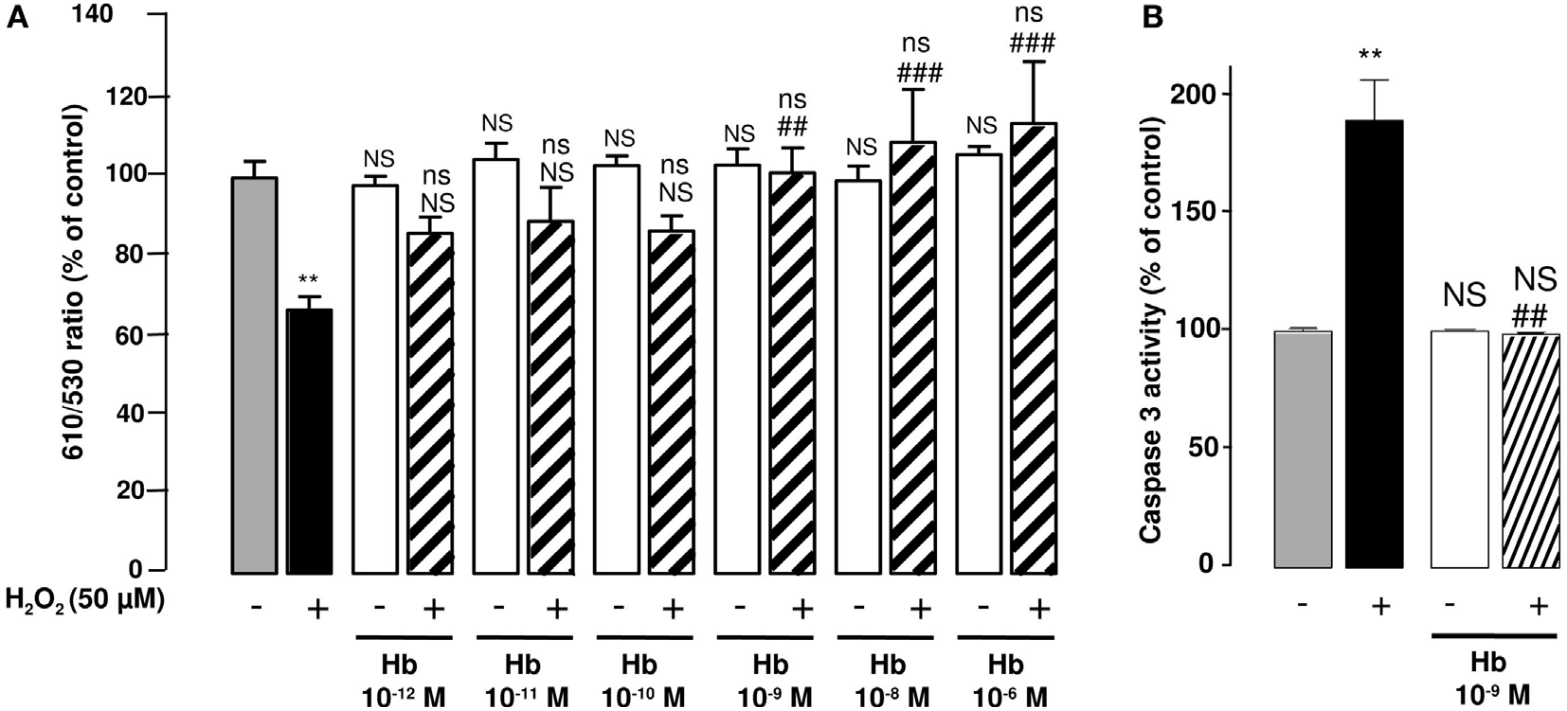
**Effect of Hb on H_2_O_2_-induced alteration of mitochondrial membrane potential and caspase-3 activation in cultured astrocytes**. Cells were co-incubated for 24 h with medium alone (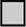) or with H_2_O_2_ (50 µM) in the absence (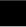) or presence of graded concentrations of Hb (10^−12^ to 10^−6^ M, 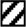). **(A)** Mitochondrial transmembrane potential was assessed by using the JC-10 probe, and the ratio of fluorescence emissions 610/530 nm was measured as an index of mitochondrial activity. **(B)** Caspase 3 activity was measured by caspase substrate, Z-DEVDRhodamine 110, fluorescence. The results are expressed as percentage of controls. Data are means ±SEM of three independent experiments. ANOVA followed by Bonferroni’s test. **p < 0.01; ns, not statistically different from control cells. ^##^*p* < 0.01; ^###^*p* < 0.001; ns, not statistically different vs. H_2_O_2_-treated cells.

All experiments were performed on DIV 5–7 astroglial cells grown up in culture medium containing 10% of FBS. After removal of medium, cells were incubated at 37°C with fresh serum-free culture medium in the absence or presence of the test substances for 24 h. Previous data indicated that incubation of astrocytes during 24 h with 50 µM H_2_O_2_ induces about 40% of cell death (36). Thus, a dose of 50 µM H_2_O_2_ was used in the present study to evaluate the effect of Hb on astrocyte survival. On the basis of the results shown in Figures 1, 2 and 3A, a concentration of 10^−9^ M Hb, which prevents the deleterious effects of H_2_O_2_, was used in all subsequent experiments. It has been previously demonstrated that 2 × 10^−5^ M H89, 10^−6^ M chelerythrine, and 10^−6^ M U0126 blocked the protective effects of some neuropeptides on cultured astrocytes (35, 37). Thus these concentrations were also used in the present study.

### Measurement of Cell Survival

At the end of the treatment period, cells were washed twice with phosphate-buffered saline (PBS, 0.1 M, pH 7.4) and incubated for 8 min with 15 µg/mL FDA-AM in the dark, rinsed twice with PBS, and lysed with a Tris/HCl solution containing 1% sodium dodecyl sulfate. Fluorescence was measured with excitation at 485 nm and emission at 538 nm using a fluorescence microplate reader FL800TBI (Bio-Tek Instruments, Winooski, VT, USA). Experiments were carried out on 16 different wells from four independent experiments.

### Measurement of Intracellular ROS Formation

ROS were detected by measuring the fluorescence of 2′,7′-dichlorofluorescein (DCF), which is derived from the deacetylation and oxidation of the non-fluorescent compound DCFH_2_-DA. At the end of the treatment period, cells were washed twice with PBS, and then incubated with 10 µM DCFH_2_-DA for 30 min at 37°C in the dark. Fluorescence was measured with excitation at 485 nm and emission at 538 nm using a fluorescence microplate reader. Experiments were carried on 18 different wells from three independent experiments.

### Measurement of NO Accumulation

Relative changes in cytosolic NO concentration in astrocytes were monitored using the fluorescent probe 4,5-diamino fluorescein diacetate (DAF). At the end of the treatment period, cells were washed twice with PBS and then incubated with 10 µm DAF-FM diacetate for 30 min. Fluorescence was measured with excitation at 485 nm and emission at 538 nm using a fluorescence microplate reader. Experiments were carried out on 18 different wells from three independent experiments.

### Measurement of Mitochondrial Activity

Mitochondrial membrane potential was quantified using the JC-10 probe. Cells seeded into 96-well plates were incubated in the absence or presence of H_2_O_2_ with or without Hb. At the end of the treatment, astrocytes were incubated in the presence of the JC-10 probe (10 µg/mL) at 37°C for 1 h, and then washed twice with PBS. In healthy astrocytes, the intact membrane potential allows the lipophilic dye JC-10 to enter into the mitochondria where it aggregates and produces an intense orange signal. In dead cells, mitochondrial membrane potential collapses so that the monomeric JC-10 probe remains cytosolic and emits a green signal. Fluorescence intensity was measured with fluorescence microplate reader and expressed as a ratio of the emission at 610 nm (orange) over 530 nm (green) to evaluate mitochondrial integrity (38). Experiments were carried out on 12 different wells from three independent experiments.

### Measurement of Caspase 3 Activity

The effect of Hb on H_2_O_2_-induced increase of caspase 3 activity was measured by using Apo-ONE Homogeneous Caspase-3/7 kit (Promega). At the end of the incubation, 100 µL of the cell suspension was incubated with 100 µL of kit buffer and caspase substrat. Caspase-3 activity was calculated from the slope of the fluorescence measured every 15 min for 3 h with excitation at 485 nm and emission at 530 nm. Experiments were carried out on 12 different wells from three independent experiments.

### Cell Cytotoxicity Measurement

The cytotoxicity of H_2_O_2_ on astrocytes was determined by measuring the activity of LDH released into the culture medium, using a LDH assay kit (Bio-Maghreb, Tunis, Tunisia) according to the manufacturer’s instructions. LDH activity was measured at 340 nm with a spectrophotometric microplate reader. The results were expressed as a percentage of total LDH release after cell lysis with 1% Triton X-100 in PBS. Experiments were carried out on 16 different wells from four independent experiments.

### Statistical Analysis

The data were presented as mean ± SEM of at least three independent experiments (*n* ≥ 3). The statistical analysis of the data was performed using Student’s *t-*test for single comparisons and by ANOVA followed by Bonferroni’s test, and two-way ANOVA test for multiple comparisons with the GraphPad software (La Jolla, CA, USA). In all cases a *p*-value of 0.05 or less was considered as statistically significant.

## Results

### Protective Effect of Hb against H_2_O_2_-Induced Astroglial Cell Death

Incubation of cultured astrocytes with 50 µM H_2_O_2_ for 24 h induced a decrease (−37.2 ± 1.9%; *p* < 0.001) of the proportion of surviving cells. Administration of graded concentrations of Hb (10^−12^ to 10^−6^ M), which did not affect cell viability by themselves, dose-dependently prevented cell death induced by 50 µM H_2_O_2_ (Figure 1A). Examination of cultures by phase-contrast microscopy revealed that H_2_O_2_ induced cell shrinkage and disturbance of the astrocyte network with appearance of retracted processes (Figure 1B, b). The effect of H_2_O_2_ on morphological alterations was slightly attenuated with low concentration (10^−12^ M) of Hb (Figure 1B, c) and totally prevented with higher concentrations of Hb, condition in which cells exhibited a flat polygonal morphology similar to that of untreated-astrocytes (Figure 1B, a,d).

### Effect of Hb on H_2_O_2_-Induced Intracellular ROS and NO Accumulation in Cultured Astrocytes

To examine whether Hb could block H_2_O_2_-induced intracellular ROS accumulation, astrocytes were labeled with CMH_2_DCFDA, which forms the fluorescent DCF compound by oxidation with ROS. Incubation of cultured astrocytes with 50 µM H_2_O_2_ alone for 24 h, induced an increase in DCF fluorescence intensity (+127 ± 18%; *p* < 0.001). Incubation of cells with graded concentrations of Hb (10^−12^ to 10^−6^ M) had no effect on DCF fluorescence intensity (Inset), but reduced in a dose-dependent manner, the effect of H_2_O_2_ on DCF formation (Figure 2A). Since large amounts of NO are produced by cells under oxidative stress status (39), we have investigated the effect of Hb on H_2_O_2_-induced NO formation in astroglial cells. By using the DAF-FM probe, which forms the fluorescent DAF compound upon oxidation by NO, we found that H_2_O_2_ induced a significant increase (+86.6 ± 18.23%; *p* < 0.001) of NO production and, in the same range of concentrations, Hb was also able to reduce in a dose-dependent manner the effect of 50 µM H_2_O_2_ on DAF formation (Figure 2B). In contrast, all concentrations of Hb tested did not affect, by themselves, NO production (inset).

### Effect of Hb on H_2_O_2_-Induced Mitochondrial Potential Alteration and Caspase-3 Activation

Considering the major action of oxidative stress in the alteration of mitochondria functions, we have examined the effect of Hb on the integrity of mitochondria by using the fluorescent ratiometric probe JC-10. Treatment of astrocytes with 50 µM H_2_O_2_ alone induced a significant reduction (−32.6 ± 3.4%; *p* < 0.01) of the 610/530 nm ratio, indicating that the mitochondrial integrity was impaired by oxidative stress. Incubation of cells with graded concentrations of Hb (10^−12^ to 10^−6^ M) did not affect 610/530 nm fluorescence ratio, but dose-dependently reduced the deleterious effect of H_2_O_2_ on the mitochondrial membrane potential, and the 610/530 nm ratio was restored to control values for concentrations greater than 10^−9^ M (Figure 3A). H_2_O_2_-induced reduction of membrane potential was associated with an increase of caspase 3 activity (+ 89.3 ± 17.19%; *p* < 0.05). Addition of Hb (10^−9^ M) in the incubation medium totally suppressed the stimulatory effect of H_2_O_2_ on caspase 3 activation (Figure 3B).

### Identification of the Signal Transduction Pathways Involved in the Glioprotective Effect of Hb on H_2_O_2_-Induced Astroglial Death

**Figure 4:**
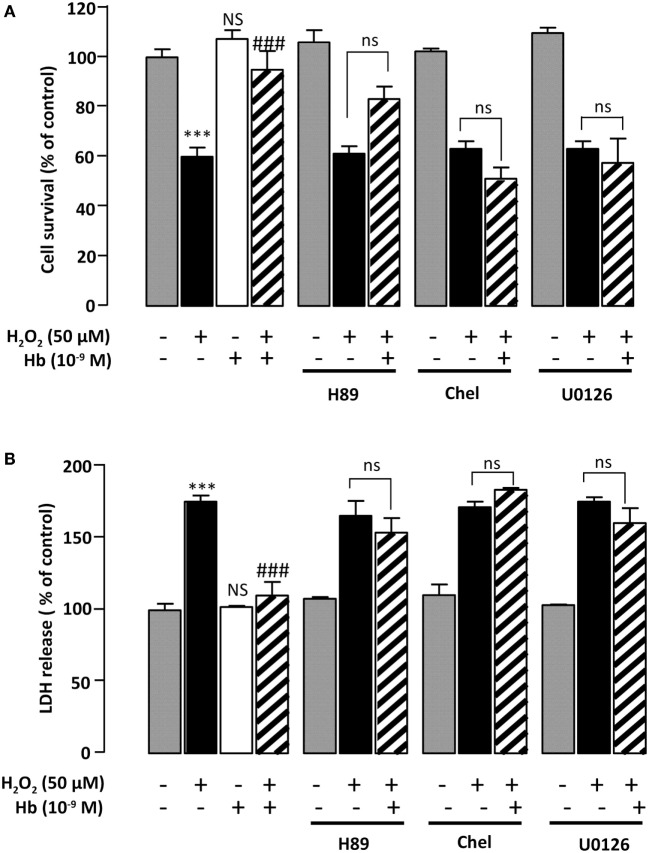
**Characterization of intracellular pathways involved in the protective effect of Hb on astroglial cells**. Cells were pre-incubated for 30 min in the absence or presence of H89 (2 × 10^−5^ M), chelerythrine (10^−6^ M; Chel), or U0126 (10^−6^ M) and then incubated for 24 h with medium alone (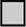) Hb (10^−9^ M) alone or with H_2_O_2_ (50 µM) in the absence (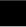) or presence of Hb (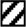
**). (A)** Cell death was determined by measuring LDH activity in culture media, and the results are expressed as percentage of LDH released in Triton-lysed cells. **(B)** Cell survival was quantified by measuring FDA fluorescence intensity, and the results are expressed as percentages of control. Data are means ± SEM of four independent experiments. ANOVA followed by Bonferroni’s test ***p < 0.001; NS, not statistically different from control cells. ^###^*p* < 0.001; ns, not statistically different vs. H_2_O_2_-treated cells.

Incubation of astrocytes with 50 µM H_2_O_2_ for 24 h induced an increase of LDH levels in the culture medium (+82.6 ± 7.55%; *p* < 0.01) (Figure 4A). Addition of Hb at the dose of 10^−9^ M to the culture medium abolished the effect of H_2_O_2_ on LDH leakage (100.2 ± 0.9%) (Figure 4A). To investigate the signaling cascade involved in the protective action of Hb, we have used pharmacological inhibitors of adenylyl cyclase (AC)/protein kinase A (PKA), phospholipase C (PLC)/protein kinase C (PKC), or mitogen-activated protein kinase (MAPK) transduction pathways. Incubation of astrocytes with the PKA inhibitor H89 (2 × 10^−5^ M), the PKC inhibitor chelerythrine (10^−6^ M) or the mitogen-activated protein kinase kinase (MEK) inhibitor U0126 (10^−6^ M), which had no effect by themself on cell damage and cell death induced by H_2_O_2_, abrogated the protective action of Hb (10^−9^ M) on H_2_O_2_-provoked toxicity and cell death (Figures 4A,B).

## Discussion

The main finding of the present study is to demonstrate for the first time that Hb protects astroglial cells against oxidative stress and death induced by H_2_O_2_ exposure. It has been found that Hb is effective at very low concentrations and exerts its glioprotective effect through inhibition of ROS and NO generation, mitochondrial dysfunctions, and caspase-3 activation.

In agreement with previous reports (35, 40, 41), we observed that H_2_O_2_-treated astrocytes exhibited modifications of cell morphology such as cell shrinkage and appearance of thin processes. In parallel, H_2_O_2_ induced an increase of LDH in the medium and a decrease of cell survival. Here, we showed that Hb dose-dependently prevented H_2_O_2_-induced cell death and abolished H_2_O_2_-evoked morphological changes. These data could be seemed in contradiction with previous studies indicating that Hb released from red blood cells in intracerebral hemorrhage is neurotoxic (9, 30, 42–44). Nevertheless, it can be noted that the concentration of Hb detected in this pathology is 10,000 times higher than that used in the present study. In addition, the concentration of Hb needed to prevent the deleterious effects of H_2_O_2_ on astroglial cells (the present data) was in the same range as that produced by neuronal cells brain, i.e., cortical and hippocampal neurons and astrocytes (25, 45, 46). Furthermore, there is now mounting evidence indicating that endogenous neuronal Hb is not detrimental in brain diseases (30) and even may exert beneficial effects on neuronal cells in *in vivo* and *in vitro* models of ischemia (24, 45). While, the precise roles of Hb in brain physiology and in neurodegenerative diseases are not well understood. Since, astrocytes produce neuroprotective compounds, that Hb protects glial cells against moderate injuries could be benefit for brain neuronal populations. As in the case of Hb, the potency and efficacy of others oxygen-binding globins, neuroglobin, and myogolobin, in preventing neuronal cell death have been proven against various neurotoxins, including H_2_O_2_, and brain injuries induced by hypoxia, ischemia, or stroke (30, 47–51). It is noteworthy that subnanomolar concentrations of Hb were still effective to protect astrocytes from the cytotoxic effect of H_2_O_2_ after 24 h of treatment, indicating that this globin exhibits a strong cytoprotective activity.

It is well documented in numerous cell types including astrocytes that H_2_O_2_ exerts its cytotoxic effect *via* the production of highly reactive species inside the cell (34, 51–53). The present study reveals that treatment of astrocytes with Hb reduced, in a concentration-dependent manner, intracellular ROS and NO levels, phenomenum, which is likely responsible for a reduction of cell death induced by H_2_O_2_. In agreement with the fact that Hb may protect astrocytes from oxidative stress, it has been demonstrated that (i) Hb strongly scavengers ROS and NO (6), (ii) Hb overexpression prevents H_2_O_2_-induced ROS surproduction and cell death in primary rat mesangial cells (54), and (iii) increasing expression of neuronal Hb prior to hypoxia insult enhances neuronal cell viability by decreasing oxidative stress process (45, 55). It cannot be excluded that peroxidase activity of Hb (56, 57) could be in part responsive for its protective effect against H_2_O_2_.

It is widely accepted that ROS can induce cell death by multiple intracellular mechanisms including mitochondrial dysfunction leading to the formation of mitochondrial permeability transition pores and thus activation of caspases, the effectors of apoptotic cell death (37, 58, 59). In agreement with this notion, measurement of mitochondria activity, with the membrane potential-sensitive probe JC-10, revealed that treatment of astrocytes with H_2_O_2_ resulted in a decrease of the proportion of active mitochondria, and that co-administration of nanomolar concentration of Hb prevented the deleterious effect of H_2_O_2_ on mitochondria. Concurrently, Hb suppressed stimulation of caspase-3/7 activity induced by H_2_O_2_. That Hb exerts its glioprotective effect through the intrinsic mitochondrial pathway is supported by a study showing that Hb blocks the effects of NO on (i) the decrease of procaspase-2 protein levels, an inactive form of caspase-2, (ii) the stimulation of caspase-3 activity, (iii) the cleavage of poly (ADP-ribose) polymerase (PARP) and the DNA fragmentation, and (iv) the apoptotic cell death in human neuroblastoma SH-SY5Y cells (60, 61). In addition, Hb reverses heme oxygenase-1/CO-provoked neuronal cell death through inhibition of caspase-3-dependent apoptotic pathway (62). Thus, collectively, these data strongly suggest that Hb rescues astrocytes from oxidative stress by preventing ROS accumulation, which in turn preserves mitochondrial activity and prevents caspase-3 activation. In support of this hypothesis, it has been shown that the globin neuroglobin, the neuropeptides octadecaneuropeptide, and pituitary adenylate cyclase-activating polypeptide, which can rescue astrocytes from oxidative damages, inhibit the effects of H_2_O_2_ on alteration of mitochondrial integrity and stimulation of caspase3/7 activity (35, 37, 51, 63).

That Hb, by itself, might peroxidate H_2_O_2_ in culture media, and that this effect might be responsible for the protective effect of Hb, cannot be totally excluded. Nevertheless, in the present study, the protective effect of Hb was shown at very low doses (10^−11^ to 10^−6^ M) and peroxidation of H_2_O_2_ by Hb has been found at doses 1,000- to 10,000 times higher than that used in the present study (56, 57). In addition, the peroxidase activity of Hb is optimal at pH 5.5, and dramatically falls down at physiological pH (57).

Although our understanding of the precise signaling pathways that trigger apoptosis of neuronal cells is still fragmentary, it has been demonstrated that activation of the AC/PKA and the PLC/PKC transduction pathways contributes to reduce intracellular ROS levels and to protect cells against oxidative stress-induced apoptosis in cultured neurons and astrocytes (34, 64, 65). Here, we show that treatment of astrocytes with the PKA inhibitor H89 or the PKC inhibitor chelerythrine abrogated the effects of Hb on H_2_O_2_-evoked caspase-3/7 activation and cell death apoptosis. We have previously demonstrated that the MAPK signaling cascade is involved in apoptotic death of astrocytes (37, 51), and it has been shown that survival of astrocytes requires phosphorylation of extracellular signal-regulated kinase (43). Consistently, we show that the protective action of Hb on astrocytes against H_2_O_2_ was abolished by the MEK inhibitor U0126. Collectively, these data suggest that the glioprotective activity of Hb can be accounted for by activation of the PKA, PKC, and MAPK transduction pathways. The receptor through which Hb might modulate intracellular pathways in astrocytes and exert its glioprotective effect is currently unknown. Alternatively, it can be proposed that Hb, in the same way as neuroglobin, could directly interact with G proteins and thus modulates transduction pathways (66–68). However, such hypothesis raises the question how Hb crosses the membrane. The delivery of Hb inside astrocytes might result to its interaction with the scavenger receptor CD163, which acts as a Hb transporter in macrophages (69). That CD163, or other receptor or mechanism, might be involved in the uptake of Hb by astrocytes merits further investigations.

The protective effect of low doses of Hb on astroglial cells might have a physiopathological significance in neurological disorders including neurodegenerative diseases, like ischemia and stroke. Clinical studies have shown that the neuronal Hb expression is reduced in brain of patient with Alzheimer disease, Parkinson disease, or dementia with Lewy Bodies (27). Along these lines, it has been reported that treatment of rat with rotenone, neurotoxin used to reproduce features of Parkinson’s disease (70), induces downregulation of Hb expression in selected neuronal populations, i.e., nigral, cortical, and striatal neurons, associated with an elevation of oxidative stress damages and mitochondria dysfunction (55, 71). In contrast, upregulation of neuronal expression of Hb is accompanied by enhanced resistance to oxidative stress under hypoxic conditions (45). Despite their high antioxidative activities, astroglial cells cannot survive and protect neurons under insurmountable oxidative stress (32). Thus, upregulation of Hb expression in astrocytes might increase protection of cells from oxidative insults and might delay neuronal damages in various cerebral injuries involving oxidative neurodegeneration.

In conclusion, the present study demonstrates that Hb at low concentrations acts as endogenous protective agent against oxidative and nitrosative stress inducing apoptosis of cultured astrocytes. The antiapoptotic effect of this globin is attributable, at least in part, to the reduction of ROS formation, which preserves mitochondrial functions and prevents caspase 3 activation.

## Ethics Statement

Approval for these experiments was obtained from the Medical Ethical Committee for the Care and Use of Laboratory Animals of Pasteur Institute of Tunis (approval number: FST/LNFP/Pro152-012).

## Author Contributions

FA, MA, and OM-K conceived and designed the experiments. FA, IG, and OM-K performed the experiments. FA, M-CT, MA, and OM-K analyzed the data. FA, IG, M-CT, MA, and OM-K contributed to reagents/materials/analysis tools. FA, M-CT, and OM-K wrote the paper.

## Conflict of Interest Statement

The authors declare that the research was conducted in the absence of any commercial or financial relationships that could be construed as a potential conflict of interest.

## Abbreviations

Hb, hemoglobin; H_2_O_2_, hydrogen peroxide; MAPK, mitogen-activated protein kinase; NO, nitric oxide; PKA, protein kinase A; PKC, protein kinase C; ROS, reactive oxygen species.
